# Impact of exercise training on tumour‐infiltrating T cells in human prostate cancer: A secondary analysis of a randomized controlled trial (PRO‐TEST)

**DOI:** 10.1113/EP092374

**Published:** 2025-02-20

**Authors:** Simon Nørskov Thomsen, Emil Wriedt, Marianne Gardar Stærk, Sissal Sigmundsdottir Djurhuus, Birgitte Grønkær Toft, Sabrina Wielsøe, Andreas Røder, Thomas Hasselager, Peter Busch Østergren, Henrik Jakobsen, Klaus Brasso, Jesper Frank Christensen, Louise Lang Lehrskov, Casper Simonsen

**Affiliations:** ^1^ Centre for Physical Activity Research Copenhagen University Hospital – Rigshospitalet Copenhagen Denmark; ^2^ Department of Endocrinology Hvidovre University Hospital Hvidovre Denmark; ^3^ Department of Pathology Copenhagen University Hospital – Rigshospitalet Copenhagen Denmark; ^4^ Department of Urology, Copenhagen Prostate Cancer Center Copenhagen University Hospital – Rigshospitalet Copenhagen Denmark; ^5^ Department of Clinical Medicine University of Copenhagen Copenhagen Denmark; ^6^ Department of Pathology Copenhagen University Hospital – Herlev and Gentofte Copenhagen Denmark; ^7^ Department of Urology Copenhagen University Hospital – Herlev and Gentofte Copenhagen Denmark; ^8^ Department of Oncology Copenhagen University Hospital – Herlev and Gentofte Herlev Denmark

**Keywords:** exercise, prostate cancer, T cells

## Abstract

Exercise training reduces tumour growth by increasing tumour‐infiltrating T‐cell density in preclinical models. However, it remains unknown whether exercise training can modify intratumoural T cells in humans.The aim of this study was to compare the effects of an exercise training intervention versus control on human prostate intratumoural T‐cell density.This study is a secondary analysis of a randomized controlled trial. We randomly allocated men (age > 18 years) with treatment‐naive localized prostate cancer scheduled for radical prostatectomy 2:1 to exercise training intervention or control. The exercise intervention consisted of supervised, high‐intensity interval bicycling four times per week from the time of randomization until prostatectomy. Intratumoural CD3^+^ and CD8^+^ T‐cell densities in diagnostic biopsies and postsurgical prostatectomy specimens were quantified using immunohistochemistry. Between‐group differences in changes from baseline to follow‐up were estimated using constrained baseline linear mixed‐effect models.A total of 30 participants were included (exercise intervention, *n* = 20; control, *n* = 10). We found no between‐group differences in changes in CD3^+^ T cells [mean difference (95% confidence interval): −17 (−185; 150) cells/mm^2^] or CD8^+^ T cells [mean difference (95% confidence interval): −16 (−206; 172) cells/mm^2^]. Additionally, we found no statistically significant correlations between changes in T‐cell density and the number of exercise training sessions attended or changes in maximal oxygen consumption.In this secondary analysis of a randomized controlled trial, we found no impact of the exercise regimen on tumour‐infiltrating CD3^+^ and CD8^+^ T‐cell density in human prostate cancer.

## Abstract (highlights)

- **What is the central question of this study?**
  Does exercise training increase human prostate intratumoural T‐cell density?
- **What is the main finding and its importance?**
  In this randomized controlled trial, we found no impact of the exercise training regimen on CD3^+^ and CD8^+^ T‐cell density. Although this suggests that exercise training might not modify intratumoural T‐cell composition in prostate cancer, important methodological challenges might limit the interpretation of our data. More studies are needed to evaluate the capacity of exercise training to modify human intratumoural immune cell composition.

## 1 INTRODUCTION

Higher levels of postdiagnosis physical activity are associated with improved cancer‐specific survival across multiple types of cancers (Friedenreich et al., 2020; McTiernan et al., 2019). However, the potential anticancer effects of exercise underpinning these associations remain largely unknown. This knowledge gap limits rational design of effective clinical exercise training regimens for patients with cancer (Iyengar & Jones, 2019).

To this end, exercise‐induced mobilization, activation and redistribution of cytotoxic immune cells is emerging as a key candidate anticancer mechanism (Fiuza‐Luces et al., 2024; Hojman et al., 2018). Animal models have shown that immune cells mobilized into the circulation during exercise can infiltrate tumours in distinct tissues, leading to suppressed tumour growth (Fiuza‐Luces et al., 2024; Hojman et al., 2018). Indeed, our group has demonstrated that voluntary wheel running reduces tumour growth through increased tumour infiltration of natural killer (NK) cells via an interleukin‐6‐dependent mechanism in mice (Pedersen et al., 2016). Following this seminal discovery, an increased interest has been directed towards the impact of exercise on intratumoural T cells. For instance, in a murine model, Gomes‐Santos et al. (2021) demonstrated that exercise‐induced reductions in tumour growth were accompanied by increased densities of intratumoural CD8^+^ T cells. Notably, administration of CD8 antibodies completely abrogated the reduction in tumour growth, suggesting that remodulation of the tumour‐infiltrating T‐cell composition plays a causal role in the anticancer effects of exercise (Gomes‐Santos et al., 2021). Similar findings have been reported in several other preclinical experiments (Hagar et al., 2019; Kurz et al., 2022; Rundqvist et al., 2020). However, it remains unknown whether this intriguing antitumour effect of exercise translates to the human setting.

To translate our preclinical findings on exercise modulation of intratumoural immune cells (Pedersen et al., 2016), we recently conducted the Prostate Cancer Exercise trial (PRO‐TEST) to investigate the effects of exercise training on immune cells in human prostate cancer (Djurhuus et al., 2023). Our primary analysis revealed no effects of exercise; however, per‐protocol analyses suggested that exercise training might increase the density of tumour‐infiltrating NK cells (Djurhuus et al., 2023), providing some of the first in‐human data to suggest that exercise might directly impact human intratumoural immune cell composition.

Here, we report the findings of a secondary analysis of the PRO‐TEST trial with the primary objective to compare the density of tumour‐infiltrating CD3^+^ and CD8^+^ T cells in exercise intervention versus control conditions. The secondary objectives were to explore the relationship between changes in tumour‐infiltrating T cells and measures of intervention adherence and response.

## 2 MATERIALS AND METHODS

This study is a secondary analysis of the PRO‐TEST trial. PRO‐TEST was a multicentre, randomized controlled trial, approved by the Ethics Committee of the Capital Region of Denmark (H‐16034670) and prospectively registered at ClinicalTrials.gov (NCT02954783). The design, including disclosure and justification of post‐registration changes to the PRO‐TEST trial, has been published elsewhere (Djurhuus et al., 2023).

### 2.1 Participants

Patients eligible for participation were adult (age > 18 years) men with histologically confirmed treatment‐naive localized adenocarcinoma of the prostate scheduled for radical prostatectomy. Ineligibility criteria were as follows: other known malignancy requiring active treatment; Eastern Cooperative Oncology Group (ECOG) performance status > 1; current treatment with β‐adrenergic receptor antagonists; physical disabilities precluding physical exercise; and inability to read and understand the Danish language. The participants were recruited from the Departments of Urology at Rigshospitalet and Herlev Hospital, Copenhagen, Denmark.

### 2.2 Group allocation

The participants were randomly allocated 1:2 to either control or exercise intervention using a computer‐generated allocation sequence of permuted blocks of size 3, without stratification. The allocation sequence was concealed to the trial personnel throughout the trial period.

### Exercise group

The intervention consisted of high‐intensity bicycling four times per week for 2–8 weeks, depending on the time from randomization to prostatectomy. All exercise‐training sessions were performed on a stationary bicycle ergometer and consisted of 10 min warm‐up at 30% of peak power output (PPO) followed by four to six intervals of 1 min at a target intensity 100%–120% of baseline PPO. Each 1 min interval was separated by 3 min active rest at 30% of PPO. The number and the target intensity of the 1 min intervals were progressively increased, as follows: week 1, four intervals of 1 min at 100% of PPO; week 2, four intervals of 1 min at 110% of PPO; weeks 3 and 4, five intervals of 1 min at 120% of PPO; and weeks 5–8, six intervals of 1 min at 120% of PPO.

All sessions were supervised by a trained instructor at the Centre for Physical Activity Research (CFAS), Rigshospitalet, Copenhagen, Denmark. Reductions in intensity and duration were recorded. The intervention contained no non‐exercise components.

### 2.3 Control group

Participants allocated to the control group received standard of care, which did not include any formal exercise training. No restrictions regarding physical activity were imposed.

### 2.4 Outcome measure

The primary outcomes of this secondary analysis were tumour‐infiltrating T cells (CD3^+^ and CD8^+^). As in the primary analysis (Djurhuus et al., 2023), single‐parameter immunohistochemical analysis was performed on 3‐µm‐thick tissue sections cut from formalin‐fixed, paraffin‐embedded tissue specimens from the diagnostic core needle biopsy with the highest percentage of tumour tissue and the corresponding tumour focus in the prostatectomy specimen. The analysis was conducted using the ready‐to‐use monoclonal mouse anti‐human CD8^+^ antibody (clone C8/144B; Dako Omnis, Agilent Technologies) on the Dako Omnis automated staining platform (Agilent Technologies) and the anti‐CD3 rabbit monoclonal primary antibody (clone 2GV6; Roche Diagnostics International) on a Ventana automated staining platform (Roche Diagnostics). Both staining procedures were performed according to the manufacturer's protocol. Slides were subsequently scanned at ×20 magnification using the Hamamatsu NanoZoomer‐XR. Owing to the high number of positive lymphocytes, CD3^+^ and CD8^+^ cells were quantified digitally using Visiopharm digital pathology software (v.2023.01.4; Visiopharm A/S, Hørsholm, Denmark). Image analysis was performed using the Nuclei Detection AI (Brightfield) APP, with the output variable being the number of positive cells per millimetre squared. The entire tumour area on the selected slide was outlined manually as the region of interest for analysis. The same approach was used to quantify an equally sized, randomly chosen area of prostatic tissue, without adenocarcinoma, for each patient sample as a control. Slides without remaining tumour foci or with poor‐quality immunohistochemical staining were excluded.

### 2.5 Maximal oxygen consumption

Maximal oxygen consumption (${\dot{V}}_{O_{2}\max}$) and PPO were assessed using an exercise test to volitional exhaustion. The test was performed on a bicycle ergometer (LC4; Monark Exercise AB, Vansbro, Sweden) and was initiated with a 3 min warm‐up at 70 W, followed by 1 min workload increments of 20 W until exhaustion. Verbal encouragement was given, and pulmonary gas exchange parameters (O_2_, CO_2_ and ventilation) were measured throughout the test (COSMED Quark CPET System; COSMED Srl, Rome, Italy). The criteria for ${\dot{V}}_{O_{2}\max}$ were attainment of two of the following: an O_2_ uptake plateau (Thomsen et al., 2026); rating of perceived excretion ≥ 17 (Borg 6–20); and respiratory exchange ratio ≥ 1.1.

### 2.6 Statistical analysis

Between‐group differences in changes from baseline to follow‐up in tumour‐infiltrating CD3^+^ and CD8^+^ T cells were estimated using constrained baseline linear mixed‐effect models (Coffman et al., 2016), including identity as a random intercept and assessment time points (baseline/follow‐up) and treatment (coded as ‘0’ at baseline for both groups and as ‘0’ and ‘1’ at follow‐up for control and intervention, respectively) as fixed effects. Additionally, given the use of different tumour tissue sampling methods pre‐ and postintervention (diagnostic core needle biopsy vs. resected specimen), we compared postintervention values between the group values using Student's two‐sample *t*‐test. The primary analyses were performed in the intention‐to‐treat (ITT) population, including all allocated participants, regardless of deviations from the allocated intervention. Secondary analyses were performed in the per‐protocol (PP) population, including only participants in the intervention group with an intervention duration of ≥5 weeks and an attendance rate of ≥75%. We explored the linear correlations between: (1) ${\dot{V}}_{O_{2}\max}$ and tumour‐infiltrating CD3^+^ and CD8^+^ cells at baseline; (2) changes in ${\dot{V}}_{O_{2}\max}$ and changes in tumour‐infiltrating CD3^+^ and CD8^+^ cells; (3) the number of attended exercise training sessions and changes in tumour‐infiltrating CD3^+^ and CD8^+^ cells; (4) changes in ${\dot{V}}_{O_{2}\max}$ and postinvention tumour‐infiltrating CD3^+^ and CD8^+^ cells; and (v) the number of attended exercise training sessions and post‐invention tumour‐infiltrating CD3^+^ and CD8^+^ cells. The analysis was performed in R (v.1.4.1717).

## 3 RESULTS

**FIGURE 1:**
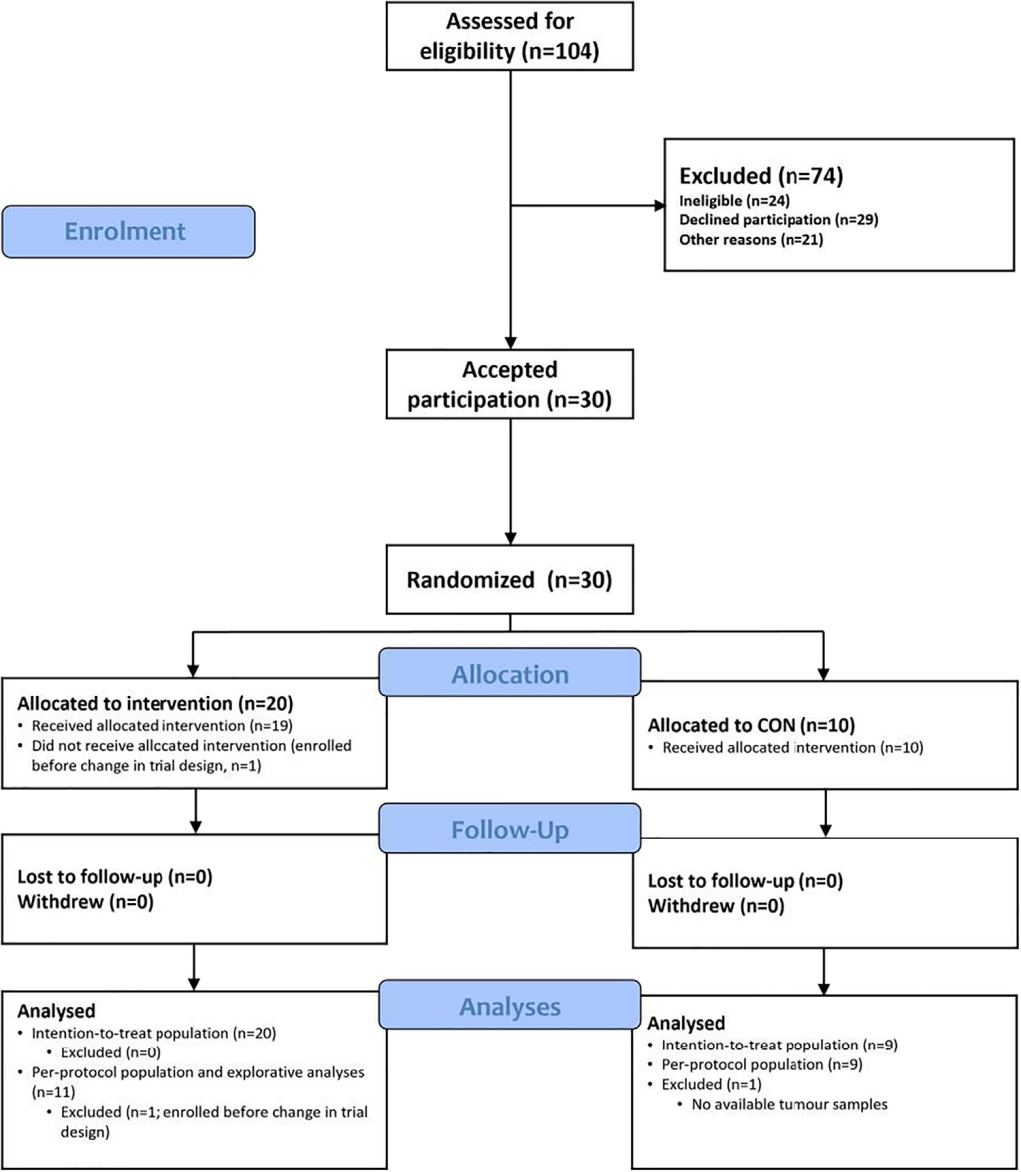
Flow chart. Abbreviation: CON, control.

We screened a total of 104 patients between November 2016 and December 2019. Of these, 80 patients were potentially eligible, and 30 (38%) consented to participate and were randomized (Figure 1). The participant baseline characteristics are presented in Table 1.

**TABLE 1:** Baseline participant characteristics.

Characteristic	Control (*n* = 10)	Intervention (*n* = 20)
Age, years, median (IQR)	68 (61–70)	63 (57–67)
BMI, kg/m^2^, median (IQR)	27 (24–30)	27 (25–29)
PSA, µg/L, median (IQR)	9.1 (8.4–18.0)	7.4 (5.4–13.0)
Gleason score, *n* (%)		
<7	2 (20)	6 (30)
7	6 (60)	15 (75)
>7	2 (20)	2 (10)
Smoking, *n* (%)		
Never	5 (50)	9 (45)
Previous	3 (30)	9 (45)
Current	2 (20)	2 (10)
Moderate–vigorous physical activity		
<150 min/week, *n* (%)	3 (30)	2 (10)
≥150 min/week, *n* (%)	7 (70)	18 (80)
Alcohol consumption, *n* (%)		
≤14 units/week	9 (90)	16 (80)
>14 units/week	1 (10)	4 (20)

Abbreviations: BMI, body mass index; IQR, interquartile range; PSA, prostate‐specific antigen.

### 3.1 Intervention adherence

The median (interquartile range) number of planned sessions was 21 (4.5), and the median (interquartile range) attendance rate was 86% (7.5%). There were no reductions in exercise training intensity or duration in any of the attended exercise training sessions. A total of 11 participants had an intervention duration ≥5 weeks and attended ≥75% of the planned sessions and were therefore included in the PP population.

### 3.2 CD3^+^ cell density

**FIGURE 2:**
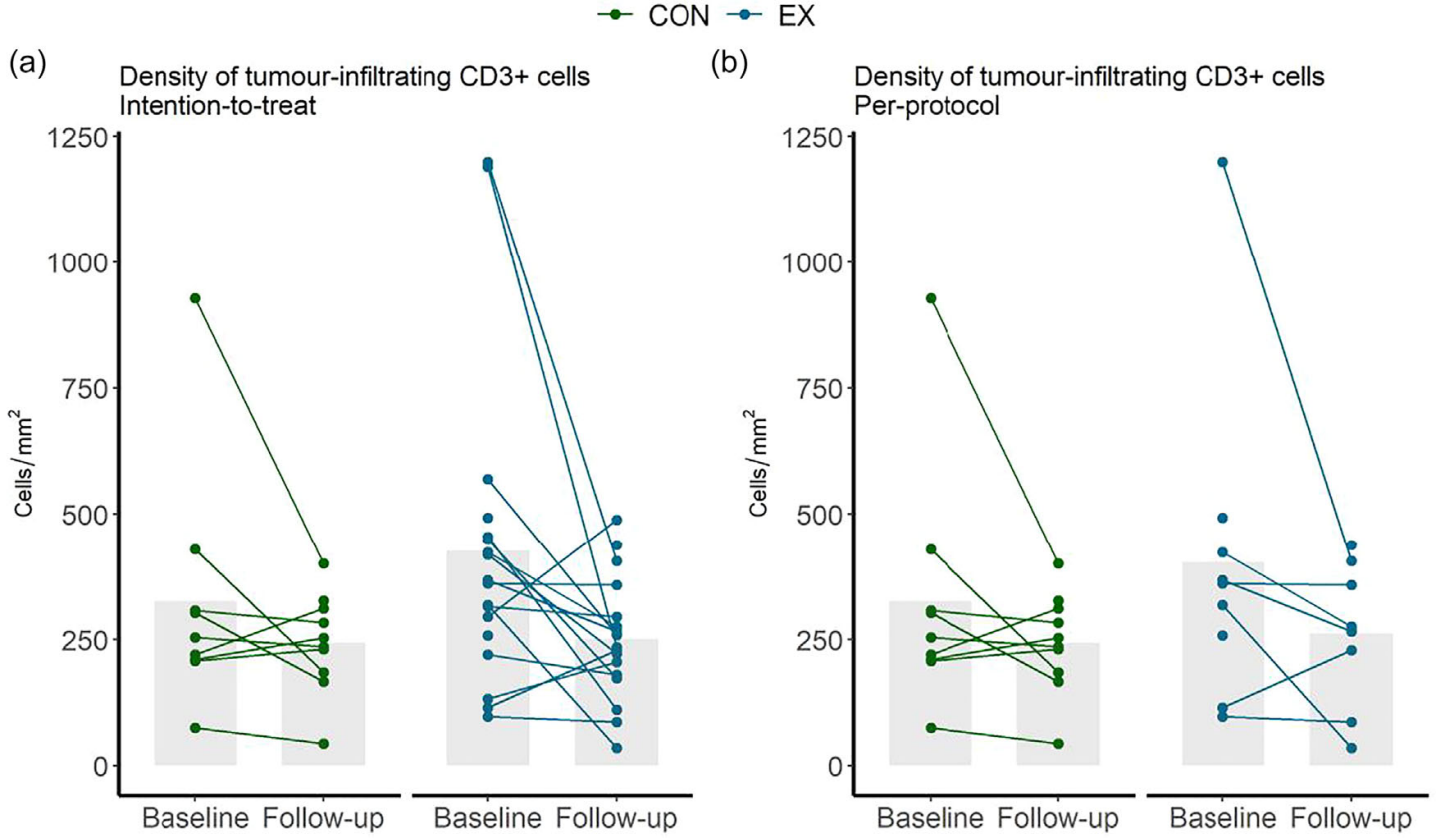
(a) Changes in tumour‐infiltrating CD3^+^ cell density in the intention‐to‐treat population. (b) Changes in tumour‐infiltrating CD3^+^ cell density in the per‐protocol population. Abbreviations: CON, control; EX, intervention.

Within‐group changes and between‐group differences in changes in CD3^+^ cells are reported in Table 2, and individual changes are presented in Figure 2. We found no between‐group difference in mean changes from baseline [mean (95% confidence interval): −18 (−196; 160) and −16 (−222; 190) cells/mm^3^ in intervention versus control in the ITT and PP populations, respectively]. Moreover, we found no difference in means at postintervention [difference in mean (95% confidence interval): 7 (−82; 96) and 18 (−112; 148) cells/mm^2^ in intervention versus control in the ITT and PP populations, respectively].

**TABLE 2:** Changes in tumour‐infiltrating T‐cell density.

	Intervention		Control		Between groups
	Baseline [mean (SD)]	Within‐group change [mean (95% CI)]	Baseline [mean (SD)]	Within‐group change [mean (95% CI)]	Difference in within‐group change [mean (95% CI)]
Intention to treat					
CD3^+^, cells/mm^2^	427 (309)	−154 (−196; −28)	326 (245)	−137 (−292; 19)	−18 (−196; 160)
CD8^+^, cells/mm^2^	174 (83)	−17 (−55; 21)	135 (76)	−32 (−86; 21)	15 (−47; 79)
Per protocol					
CD3^+^, cells/mm^2^	395 (309)	−129 (−296; 40)	326 (245)	−112 (−264; 41)	−16 (−222; 190)
CD8^+^, cells/mm^2^	189 (106)	16 (−40; 72)	135 (76)	−29 (−85; 25)	46 (−30; 121)

*Note*: *n* = 20 and *n* = 11 in the intervention group for the intention‐to‐treat and the per‐protocol analyses, respectively; *n* = 9 in both the intention‐to‐treat and the per‐protocol analyses in the control group.

Abbreviation: CI, confidence interval.

**FIGURE 3:**
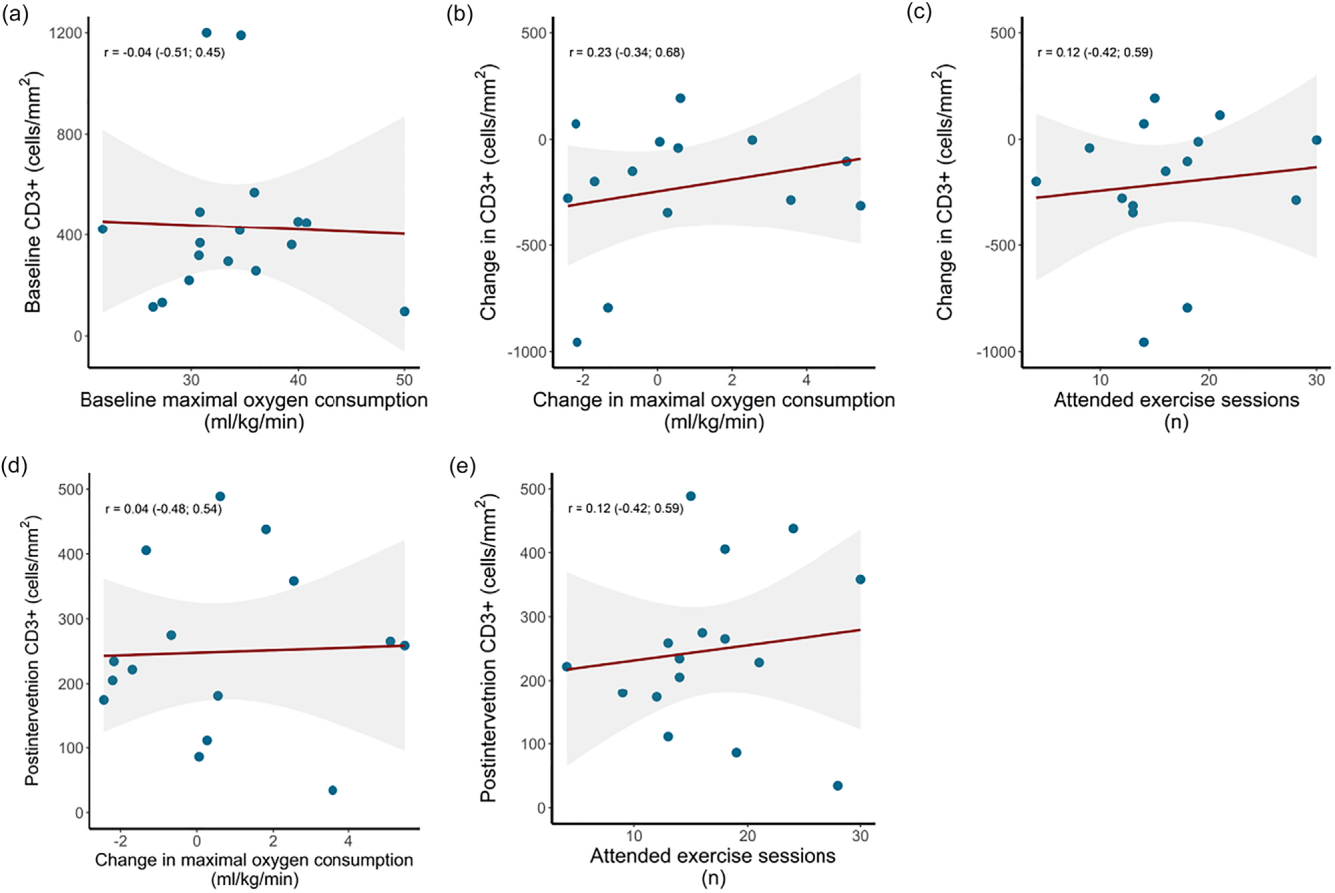
Linear correlations between: (a) tumour‐infiltrating CD3^+^ cell density and maximal oxygen consumption at baseline; (b) changes in tumour‐infiltrating CD3^+^ cell density and changes in maximal oxygen consumption; (c) changes in tumour‐infiltrating CD3^+^ cell density and number of attended exercise training sessions; (d) tumour‐infiltrating CD3^+^ cell density postintervention and change in maximal oxygen consumption; and (e) tumour‐infiltrating CD3^+^ cell density postintervention and number of attended exercise training sessions. The shaded area represents the 95% confidence intervals of the correlation coefficient. Values in parentheses are 95% confidence intervals.

There were no linear correlations in the intervention group between CD3^+^ cell density and ${\dot{V}}_{O_{2}\max}$ at baseline (Figure 3a); change in CD3^+^ density and change in ${\dot{V}}_{O_{2}\max}$ or the number of attended exercise training sessions (Figure 3b,c); or postintervention CD3^+^ density and change in ${\dot{V}}_{O_{2}\max}$ or the number of attended exercise training sessions (Figure 3d,e).

### 3.3 CD8^+^ density

**FIGURE 4:**
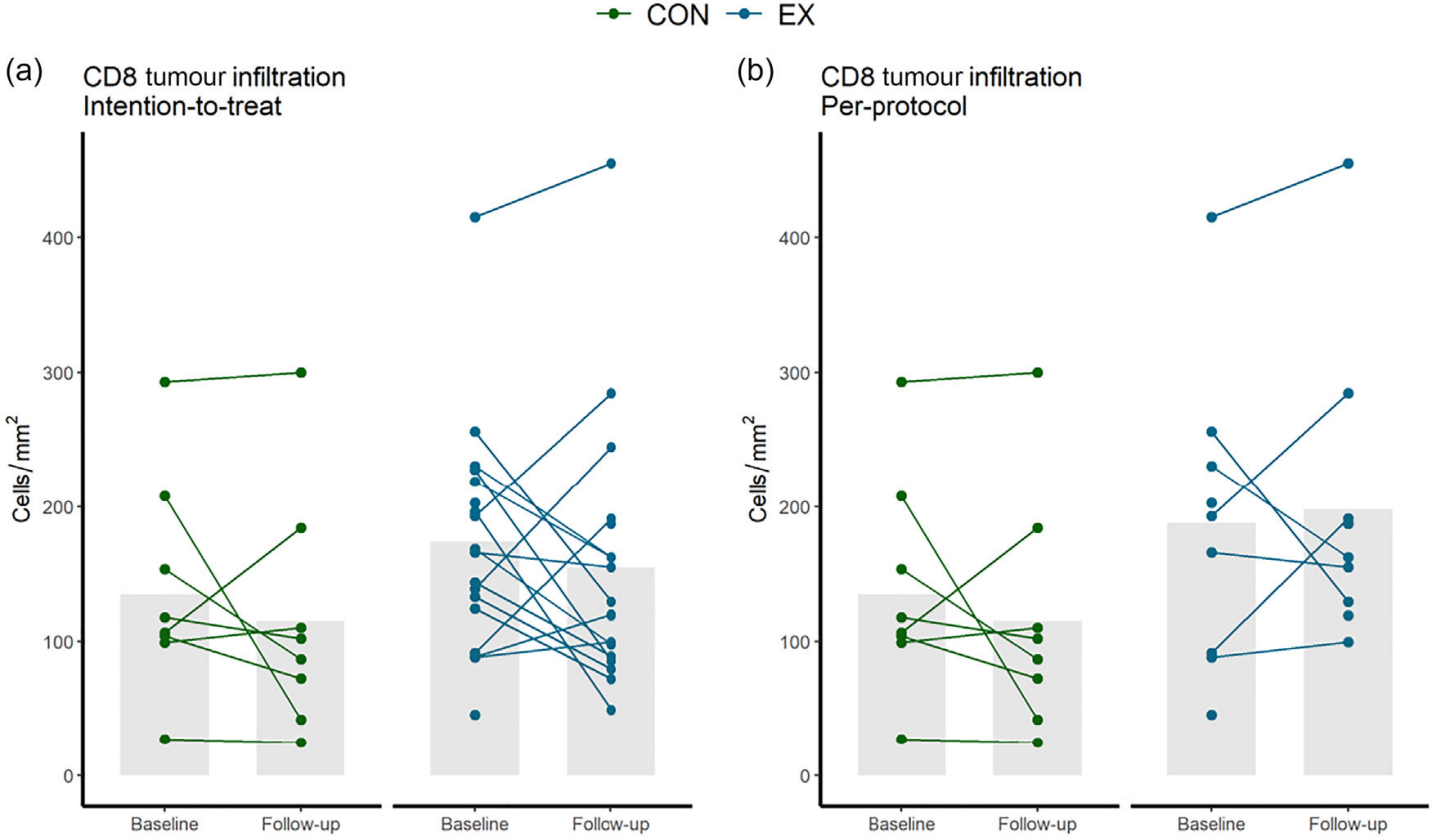
(a) Changes in tumour‐infiltrating CD8^+^ cell density in the intention‐to‐treat population. (b) Changes in tumour‐infiltrating CD8^+^ cell density in the per‐protocol population. Abbreviations: CON, control; EX, intervention.

Table 2 presents within‐group changes and between‐group differences in changes in CD8^+^ cells. Individual changes are reported in Figure 4. There was no between‐group difference in mean change from baseline [mean (95% confidence interval): −16 (−222; 190) and 46 (−30; 121) cells/mm^3^ in ITT and PP, respectively, in intervention vs. control]. Additionally, we found no difference in means at postintervention [difference in mean (95% confidence interval): 40 (−44; 122) and 83 (−20; 186) cells/mm^2^ in intervention vs. control in ITT and PP, respectively].

**FIGURE 5:**
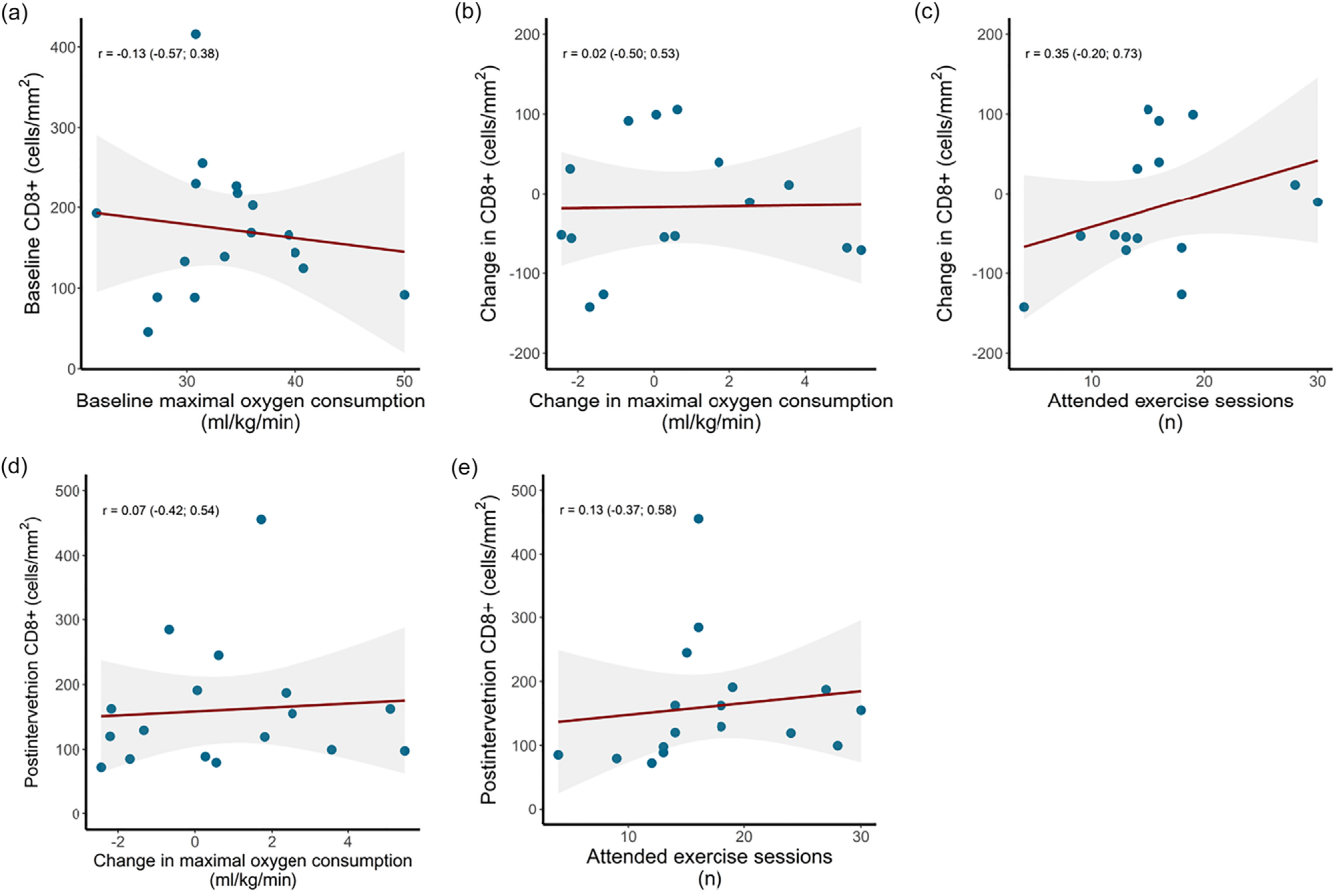
Linear correlations between: (a) tumour‐infiltrating CD8^+^ cell density and maximal oxygen consumption at baseline; (b) changes in tumour‐infiltrating CD8^+^ cell density and changes in maximal oxygen consumption; (c) changes in tumour‐infiltrating CD8^+^ cell density and number of attended exercise training sessions; (d) tumour‐infiltrating CD8^+^ cell density postintervention and change in maximal oxygen consumption; and (e) tumour‐infiltrating CD8^+^ cell density postintervention and number of attended exercise training sessions. The shaded area represents the 95% confidence intervals of the correlation coefficient. Values in parentheses are 95% confidence intervals.

In the intervention group, we found no linear correlations between CD8^+^ cell density and ${\dot{V}}_{O_{2}\max}$ at baseline (Figure 5a); change CD8^+^ density and change in ${\dot{V}}_{O_{2}\max}$ or the number of attended exercise training sessions (Figure 5b,c); or postintervention CD8^+^ density and change in ${\dot{V}}_{O_{2}\max}$ or the number of attended exercise training sessions (Figure 5d,e).

## 4 DISCUSSION

This study is, to our knowledge, the first randomized controlled trial to explore the impact of exercise training on tumour‐infiltrating T cells in humans. The principal finding was that there were no differences in the densities of CD3^+^ and CD8^+^ T cells between the exercise intervention and the control group. Thus, this study provides early evidence to suggest that structured, supervised exercise training prior to radical prostatectomy might not alter the composition of intratumoural T cells in men with prostate cancer.

The results of this study extend our prior analysis of the PRO‐TEST trial, where we observed no differences in the density of tumour‐infiltrating NK cells between the exercise intervention and control groups (Djurhuus et al., 2023). However, we did observe a within‐group increase in the exercise group in the per‐protocol analysis, and higher volumes of completed exercise training were correlated positively with larger increases in NK cell density (Djurhuus et al., 2023). These observations are in line with the general notion that NK cells are more responsive to exercise in comparison to other lymphocytes (Idorn & Hojman, 2016). For instance, characterizations of acute exercise‐induced leucocytosis in patients with cancer by our group (Schauer et al., 2022) and others (Koivula et al., 2023) demonstrate a more pronounced increase in circulating NK cells compared with CD3^+^ and CD8^+^ T cells. Additionally, the findings are in line with our previous observations in mice, where voluntary exercise training led to larger accumulation of tumour‐infiltrating NK cells compared with other lymphocyte subsets (Pedersen et al., 2016).

Contrary to our findings, a recent study by Kurz et al. (2022) concluded that exercise training prior to pancreatectomy increases intratumoural CD8^+^ T‐cell density in humans. Important methodological differences might explain these contrasting results. Kurz et al. (2022) used a non‐randomized set‐up, comparing historical controls with participants in a single‐arm exercise trial, and used postintervention tumour tissue samples to estimate the effects of exercise training. In contrast, we used a randomized controlled design, and we collected tumour tissue samples both before and after intervention. Besides these key methodological differences, direct comparison between our study and the one by Kurz et al. (2022) is challenging because of the considerable differences in the exercise training protocols and patient sample populations. We evaluated short‐term, high‐intensity interval cycling in localized treatment‐naive prostate cancer. In contrast, Kurz et al. (2022) investigated the effects of a longer‐term multimodal exercise intervention, consisting of 17 weeks of 60 min moderate‐intensity aerobic and resistance exercise in patients undergoing neoadjuvant treatment prior to pancreatectomy.

We encountered important methodological challenges that should be considered in the interpretation of our findings. In designing this trial, we considered localized prostate cancer a scientifically compelling model for exploring exercise‐induced modulation of tumour‐infiltrating immune cells owing to the ready availability of pre‐ and postintervention treatment‐naive tumour tissue samples. However, the time from randomization to prostatectomy varied considerably among the included participants, yielding large variations in the duration of the exercise training intervention (2–8 weeks). Similar variations in intervention duration were reported in a recent study that evaluated the impact of exercise intervention on human prostate tumour biology (Jones et al., 2024). Future studies seeking to explore the impact of exercise training on human tumours might benefit from using clinical settings that allow delivery of more standardized doses of exercise training.

In addition, there are several limitations to our assessments of tumour‐infiltrating T cells. First, we compared diagnostic core needle biopsy with the corresponding tumour focus in the prostatectomy specimen. Prostate tumour tissue is largely multifocal and heterogeneous in nature (Linch et al., 2017). Despite our efforts to sample from the same tumour focus, it is possible that the large variability in T‐cell density changes might partly reflect different tissue sampling techniques rather than true intervention effects. Second, by using single‐parameter immunohistochemical staining, we were not able to differentiate between CD8^+^ T‐cell subpopulations or to differentiate CD8^+^ T cells from other CD8‐expressing cells, such as NK cells and dendritic cells. This limits our estimates of CD8^+^ T‐cell density. Lastly, we did not include measures of T‐cell function, and our analysis is limited to estimates of cell density.

## 5 CONCLUSION

In conclusion, in this randomized controlled trial we found no impact of the exercise regimen on CD3^+^ and CD8^+^ T‐cell density. Although this suggest that exercise training might not modify intratumoural T‐cell composition in prostate cancer, important methodological challenges, including large variations in intervention duration, might limit the interpretation of our data. More studies are needed to evaluate the capacity of exercise training to modify human intratumoural immune cell composition.

## AUTHOR CONTRIBUTIONS

All authors have approved the final version of the manuscript and agree to be accountable for all aspects of the work. All persons designated as authors qualify for authorship, and all those who qualify for authorship are listed. Study concept, design, and protocol writing: Sissal Sigmundsdottir Djurhuus, Jesper Frank Christensen, Klaus Brasso; Data collection and acquisition: Sissal Sigmundsdottir Djurhuus, Simon Nørskov Thomsen, Sabrina Wielsøe, Thomas Hasselager, Emil Wriedt, Marianne Gardar Stærk, Birgitte Grønkær Toft; Patient recruitment: Klaus Brasso, Martin Andreas Røder, Sissal Sigmundsdottir Djurhuus, Peter Busch Østergren, Henrik Jakobsen; Statistical analysis: Simon Nørskov Thomsen, Casper Simonsen; Data analysis and interpretation: Sissal Sigmundsdottir Djurhuus, Birgitte Grønkær Toft, Jesper Frank Christensen, Casper Simonsen, Emil Wriedt, Marianne Gardar Stærk, Louise Lang Lehrskov, Klaus Brasso; Manuscript preparation: Simon Nørskov Thomsen, Louise Lang Lehrskov, Casper Simonsen; Critical review and edit of the final version of the manuscript: all authors; Project supervision: Jesper Frank Christensen, Casper Simonsen, Birgitte Grønkær Toft, Klaus Brasso.

## CONFLICT OF INTEREST

The authors declare no conflicts of interest.

## Data Availability

The data underlying our findings can be shared upon reasonable request directed to the corresponding author.
